# Biodegradable Adhesive Systems for Bio‐Integrated Applications

**DOI:** 10.1002/advs.202512633

**Published:** 2025-11-19

**Authors:** Won Bae Han, Sungkeun Han, Prashant Shivaji Shewale, Hyewon Cho, Li‐Hyun Kim, Venkata Ramesh Naganaboina, Suk‐Won Hwang

**Affiliations:** ^1^ KU‐KIST Graduate School of Converging Science and Technology Korea University Seoul 02841 Republic of Korea; ^2^ School of Electronic Engineering Kyonggi University Suwon Gyeonggi‐do 16227 Republic of Korea; ^3^ Department of Electronics and Communication Engineering, Amrita School of Engineering Amrita Vishwa Vidyapeetham Amaravati Campus Amaravati Andhra Pradesh 522503 India; ^4^ Center for Biomaterials Biomedical Research Institute Korea Institute of Science and Technology (KIST) Seoul 02792 Republic of Korea; ^5^ Department of Integrative Energy Engineering Korea University Seoul 02841 Republic of Korea

**Keywords:** adhesives, biodegradable, bioelectronics, biomedical applications, wound closure

## Abstract

Biodegradable adhesives, unlike their traditional counterparts, are engineered to bond to biological tissues while naturally degraded over time, thereby eliminating the need for removal procedures and reducing the risk of chronic inflammation. These unique features are particularly suitable for temporary biomedical applications such as wound closure, internal sealing, or integration with electronics for active/passive functions. The adhesive performance arises from the strategic combination of biodegradable polymers and adhesion mechanisms that dynamically interact with tissue surfaces. This review introduces recent advancements in biodegradable adhesives through a mechanism‐based framework, focusing on five key adhesion strategies: physical interlocking, hydrogen bonding, catechol chemistry, amine‐carboxyl coupling, and covalent bonding via diazirine or isocyanate linkages. For each strategy, representative material systems, functional properties, and biomedical implementations that enable strong, conformal adhesion under wet and physiological environments are highlighted, and with a discussion of current challenges and future directions toward intelligent, multifunctional bioadhesives for clinical uses are concluded.

## Graphical abstract

**Biodegradable adhesives** provide temporary yet reliable adhesion while degrading into safe, non‐toxic by‐products under physiological or environmental conditions. This review summarizes recent developments in physical and chemical adhesion mechanisms—including hydrogen bonding, catechol chemistry, amine‐carboxyl coupling, and emerging diazirine and urea linkages—and discusses fabrication strategies, functional performance, and future opportunities for biomedical and bioelectronic applications.

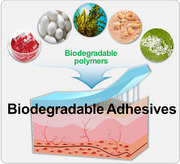

## 1 Introduction

Bioadhesives are functional materials designed to bond to biological tissues, offering critical utility across a broad spectrum of biomedical applications, including wound closure/tissue sealing,^[^ ^1^, ^2^, ^3^ ^]^ hemostasis,^[^ ^4^, ^5^, ^6^ ^]^ drug delivery,^[^ ^7^, ^8^ ^]^ and medical device fixation.^[^ ^9^, ^10^, ^11^ ^]^ The ability to conform to soft, hydrated, and often irregular tissue surfaces enables minimally invasive application, strong interfacial contact, and rapid bonding, suitable for surgical and emergency settings.

As much of the effort in biomedical research moves toward implantable and multifunctional systems, the limitations of conventional, non‐degradable adhesives—such as the need for surgical removal and the risk of chronic inflammation—have become increasingly apparent. In this context, biodegradable adhesives have emerged as a next‐generation solution, offering both robust adhesion and controlled degradation under physiological conditions.^[^ ^12^, ^13^, ^14^, ^15^, ^16^, ^17^ ^]^ These adhesives are engineered to break down into non‐toxic byproducts after fulfilling the function, which reduces the need for secondary interventions and minimizes long‐term foreign body response. Consequently, they are particularly well‐suited for temporary biomedical applications such as internal wound healing,^[^ ^18^, ^19^, ^20^, ^21^, ^22^, ^23^ ^]^ localized therapy,^[^ ^24^, ^25^, ^26^, ^27^, ^28^, ^29^ ^]^ and bioresorbable electronic interfaces.^[^ ^30^, ^31^, ^32^, ^33^, ^34^, ^35^ ^]^

**Figure 1:**
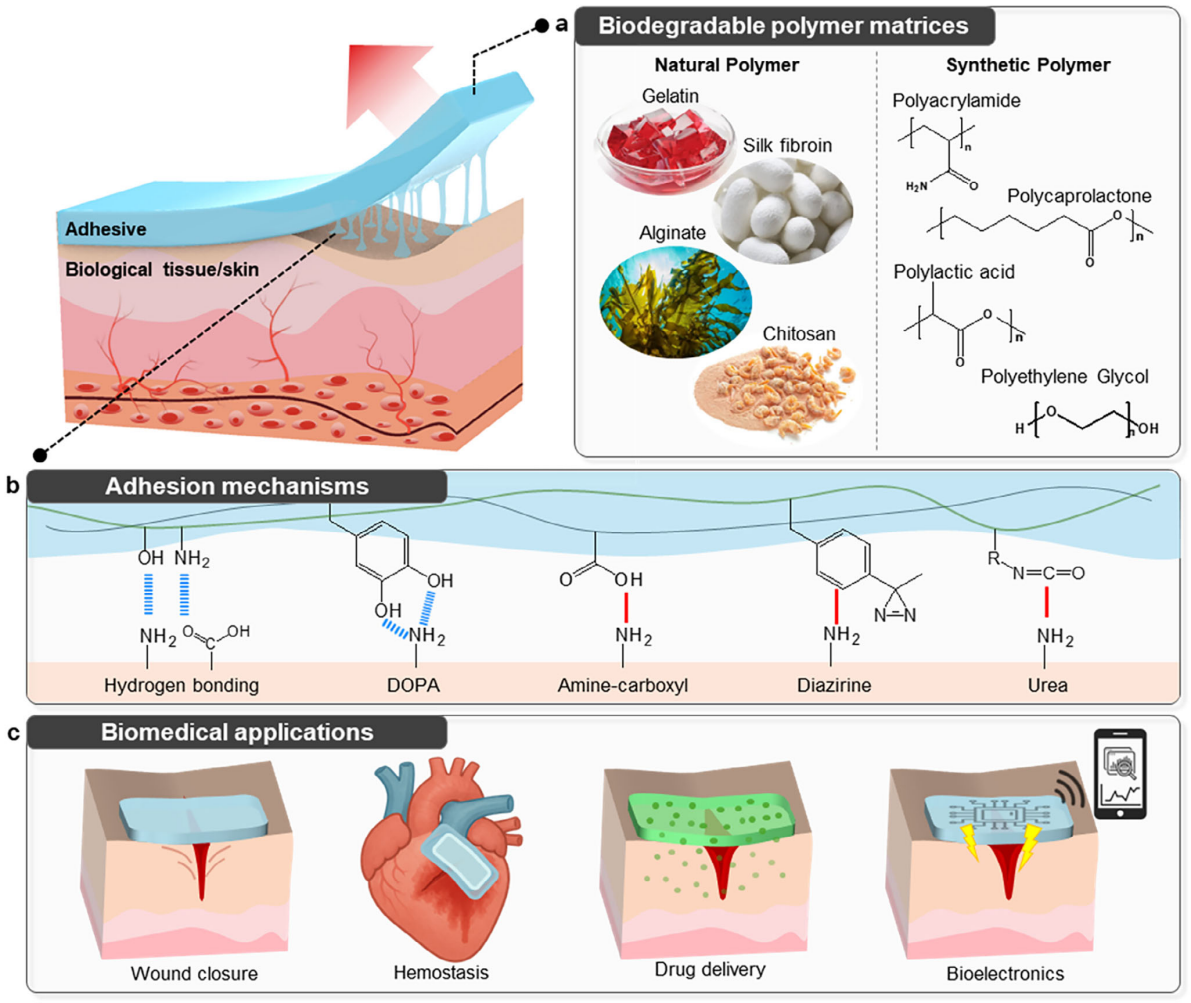
Biodegradable adhesives for biomedical applications. a) Representative natural and synthetic biodegradable polymer backbones. b) Key tissue adhesion mechanisms including hydrogen bonding, dopamine (DOPA)‐mediated interaction, amine‐carboxyl interactions, and diazirine and urea linkages. c) Biomedical applications of biodegradable adhesives: wound closure/defect sealing, hemostasis, drug delivery, bioelectronics interface, and fixation.

Characteristics of bioadhesives depend primarily on two key components: the polymer matrix and adhesion mechanism. As illustrated in **Figure** **1**, a wide range of natural polymers (e.g., gelatin, alginate, chitosan, and silk fibroin)^[^ ^36^, ^37^, ^38^, ^39^ ^]^ and synthetic biodegradable polymers (e.g., polyacrylamide, polycaprolactone, polylactic acid, and polyethylene glycol)^[^ ^40^, ^41^, ^42^, ^43^, ^44^ ^]^ serve as the structural backbone. These polymers provide tunable mechanical strength, degradation kinetics, and chemical functionality that enable both tissue compatibility and molecular‐level adhesion. Adhesion to tissues occurs through diverse mechanisms including physical interlocking with tissue microstructures,^[^ ^3^, ^16^, ^45^ ^]^ hydrogen bonding with polar groups on tissue surfaces,^[^ ^9^, ^31^, ^46^ ^]^ dopamine‐mediated catechol chemistry (DOPA),^[^ ^33^, ^47^, ^48^ ^]^ amide bonding via amine–carboxylic interactions,^[^ ^12^, ^35^, ^49^ ^]^ covalent bonding via diazirine‐derived carbene insertion,^[^ ^5^, ^29^, ^50^ ^]^ and urea linkage formation,^[^ ^51^, ^52^ ^]^ often from isocyanate precursors. Each mechanism offers distinct bonding characteristics depending on tissue type, pH, hydration, and functional group availability. Appropriate selection and integration are critical to adhesive performance in clinical and biological settings.

In this review, we categorize recent advances in biodegradable bioadhesives based on the underlying mechanisms: (1) physical adhesion, (2) hydrogen bonding, (3) catechol‐based adhesion, (4) amine–carboxylic interactions, and (5) other emerging chemistries including diazirine and urea linkages (summarized in **Table** **1**). Through this mechanism‐centered framework, we aim to highlight the material innovations, functional benefits, and biomedical potential of biodegradable bioadhesives.

**Table 1:** Comprehensive comparison of biodegradable adhesives in terms of mechanisms, materials, performance, degradation behavior, and biocompatibility.

Adhesion strategy	Primary mechanism	Materials	Adhesion strength	Applicability	Degradation (mechanism/rate)	Biocompatibility	REFs
Structural (physical)	Microtopography, capillary/viscous effects	Poly(glycerol sebacate)	≈23/ ≈20/ ≈18 kPa on heart/lung/liver (wet, shear)	High	Hydrolytic, ≈20% mass loss (5 weeks)	≈94 viability (MTT, HepG2)	[45]
Structural (physical)	Microtopography, covalent bonding	Poly(glycerol sebacate acrylate)	≈0.7 N cm^−2^ on recuts muscle	High	Hydrolytic, ≈5% mass loss (1 week)	Mild inflammation, no giant cells or fibrosis (1 week, post implantation)	[60]
Particulate (physical)	Interface bridging	Mesoporous silica nanoparticles/supraballs	≈10 J m^−2^ (dry, hydrogel‐hydrogel), ≈8 J m^−2^ (wet, hydrogel‐hydrogel)	Low	Hydrolytic, ≈90% mass loss (24 h)	–	[16]
Hydrogen bonding (chemical)	Hydrogen bonding	Gelatin, sodium pyrrolidone carboxylic acid	≈70/ ≈30/ ≈28 kPa on glass/porcine skin/leaf (dry, peel‐off)	High	Hydrolytic, fully degraded within 8 h	≈96% viability (MC3T3‐E1)	[31]
Hydrogen bonding (chemical)	Hydrogen bonding, swelling	N,N‐dimethylacrylamide, sodium alginate, N,N’‐cystaminebis(acrylamide)	≈12.33/ ≈9.30 kPa on skin/gastric tissue (wet)	High	Hydrolytic, ≈53% area decrease (8 weeks, post implantation)	≈90% viability (3T3, CCK‐8)	[27]
Hydrogen bonding (chemical)	Hydrogen bonding	Polyacrylamide, β‐cyclodextrin, 2‐(acryloyloxy)ethyltrimethylammonium chloride	≈30 kPa on skin/liver/heart (wet)	High	Hydrolytic, ≈40% mass loss (4 weeks, post implantation)	≈100% viability (3T3, metabolic assay)	[30]
Catechol‐based (chemical)	Catechol chemistry, particle bridging, NHS coupling	Dopamine hydrochloride, sodium alginate, PLGA nanoparticlesx11	≈33 kPa on porcine skin‐muscle interface (dry, shear)	High	Hydrolytic, ≈53% mass loss (4 weeks)	≈100% viability (HDFs, MTS)	[21]
Catechol‐based (chemical)	Catechol chemistry, hydrogen bonding, NHS coupling	Mussel adhesive proteins, polyacrylic acid, poly(meth)acrylic acid	≈84/ ≈105 kPa on porcine skin (wet, shear/tensile) ≈73/ ≈94 kPa on muscle (wet, shear/tensile)	High	Hydrolytic, ≈98.4/ ≈64% mass loss (2 weeks, aCUBAP and a/maCUBAP)	≈94% viability (NIH3T3, CCK‐8)	[14]
Amine‐carboxyl interaction (chemical)	Amine‐carboxyl interaction, hydrogen bonding	Polyacrylic acid‐NHS ester, poly(vinyl alcohol)	≈500 J m^−2^ on colon	High	Hydrolytic, ≈60% mass loss (12 weeks)	≈100% viability (Caco‐2, LIVE/DEAD)	[15]
Amine‐carboxyl interaction (chemical)	Amine‐carboxyl interaction	Sodium alginate, PEG‐lactide‐diacrylate, chitosan	≈300/ ≈240/ ≈80/ ≈70/ ≈50 J m^−2^ on skin/epicardium /gastrointestinal wall /renal cortex/hepatic lobule (wet)	High	Hydrolytic, ≈50% mass loss (6 weeks)	Cytocompatible (L929, confocal imaging, in vitro; H&E, GFAP/IBA1, CD68/CD163 straining, in vivo)	[35]
Amine‐carboxyl interaction (chemical)	Amine‐carboxyl interaction, hydrogen bonding, blood‐repelling	Polyacrylic acid‐NHS ester/ chitosan‐based microparticles	≈240 J m^−2^ on porcine skin (wet)	High	Gradual, macrophage‐mediated biodegradation; long‐term resorption over ≈12 weeks (no explicit rate reported)	≈98% viability (H9c2, LIVE/DEAD)	[17]
Carbene insertion (chemical)	Carbene insertion, covalent bonding	Polycaprolactone triol, diazirine	≈130 kPa on collagen/muscle, ≈800/ ≈300 kPa on Bone interior/posterior (shear)	High	Hydrolytic, fully resorbed (3 weeks, post implantation)	Biocompatible (H&E/MT histology, in vivo; blood compatibility)	[29]
Urea linkage (chemical)	Isocyanate‐thiol crosslinking, NCO‐tissue coupling	Hyperbranched polythioether, PEG‐isocyanate	≈170 kPa on porcine skin, 330–420 kPa on metals/glass	High	Hydrolytic, ≈4% mass loss (8 weeks)	≈95% viability (L929, MTT, LIVE/DEAD)	[51]

## 2 Physical Approach

Tissue adhesives face several challenges, such as strong and stable adhesion in wet, dynamic environments, while maintaining biocompatibility and mechanical durability under physiological stress. To address these limitations, structural engineering approaches have been developed to enhance adhesion via physical designs or configurations, independent of chemical functionalization.

**Figure 2:**
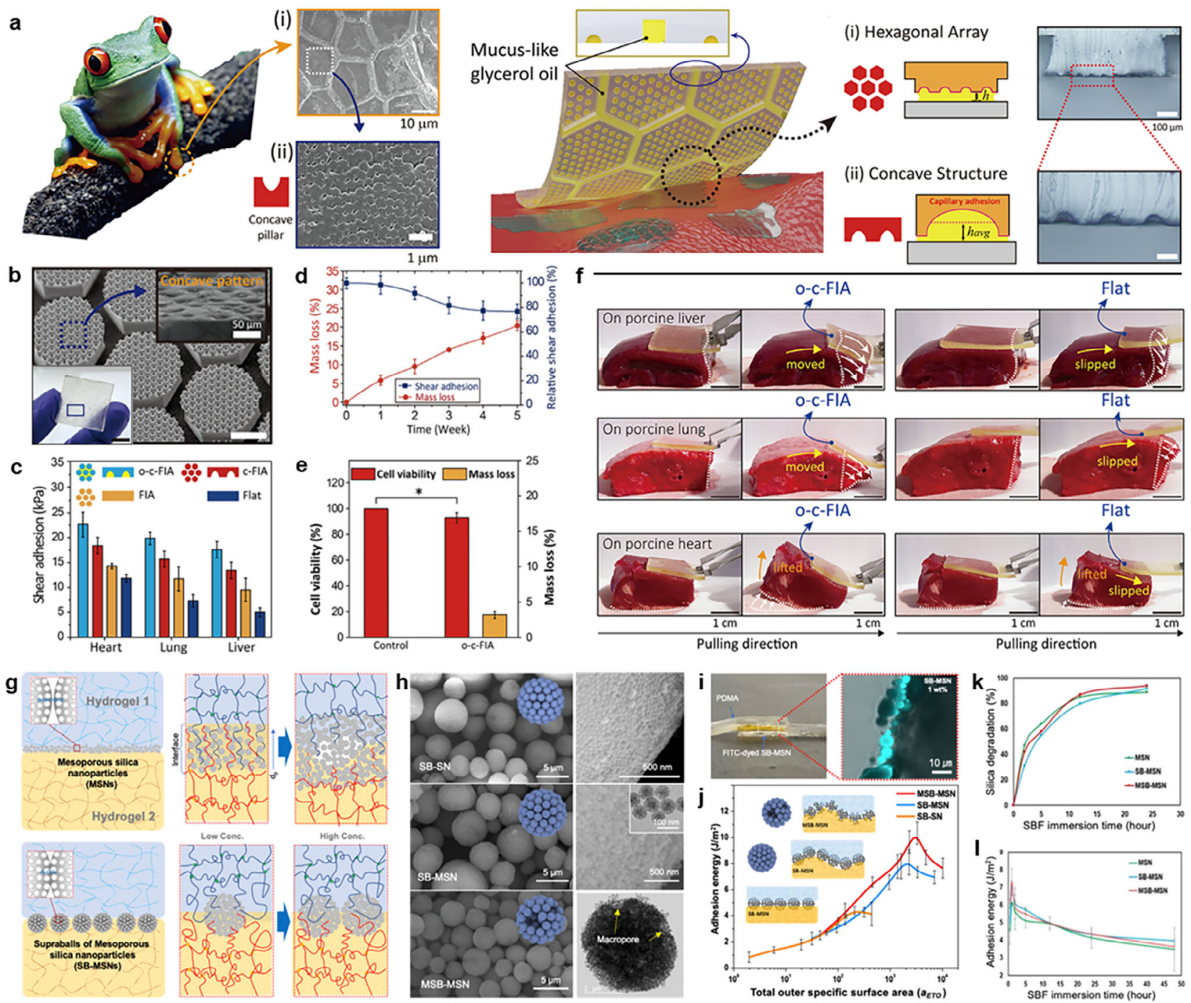
Physical adhesions. a) Scanning electron microscopy (SEM) images of hexagonal micro‐channel and concave cup structures in the toe pad of frogs (top), and schematic illustrations of a frog‐inspired adhesive film (FIA) with oil‐coated hierarchical structures featuring concave cup architectures within each hexagonal cell (bottom). b) Optical (left) and SEM (right) images of an FIA based on biodegradable poly(glycerol sebacate) (PGS). c) Comparison of shear adhesion strengths of a pristine PGS film (Flat), FIA, FIA with concave cups (c‐FIA), and oil‐filled concave cups (o‐c‐FIA) films on wet porcine liver, lung, and heart. d) Time‐dependent changes in mass and shear adhesion force of o‐c‐FIA over five weeks of immersion in phosphate‐buffered saline (PBS) at 37 °C. e) (3‐(4,5‐dimethylthiazol‐2‐yl)‐2,5‐diphenyltetrazolium bromide) (MTT) assay‐based cytocompatibility and mass loss of a Flat (Control) and o‐c‐FIA films. f) A set of optical images showing tissue detachment tests of o‐c‐FIA and flat films from porcine liver, lung, and heart. Reproduced with permission.^[^ ^45^ ^]^ Copyright 2022, Elsevier. g) Schematic illustrations of hydrogel‐hydrogel adhesion via polymer chain bridging by mesoporous silica nanoparticles (MSNs, top) and supraballs composed of partially sintered, aggregated MSNs (SB‐MSNs, bottom). h) SEM images of supraballs composed of nonporous silica nanoparticles (SB‐SNs, top), SB‐MSNs (middle), and macroporous SB‐MSNs (MSB‐MSNs, bottom), with a corresponding transmission electron microscopy (TEM) image (right). i) Optical (left) and fluorescence (right) images of poly(N,N‐dimethylacrylamide) (PDMA) hydrogels glued with fluorescently labeled SB‐MSNs. j) Adhesion energies of SB‐SNs, SB‐MSNs, and MSB‐MSNs measured as a function of the total outer specific surface area. k) Biodegradation profiles of MSN, SB‐MSN, and MSB‐MSN in simulated body fluid (SBF). l) Time‐dependent changes in adhesion energy of PDMA hydrogels bonded with MSN, SB‐MSN, and MSB‐MSN in SBF. Reproduced with permission.^[^ ^16^ ^]^ Copyright 2022, American Chemical Society.

Among various organisms capable of achieving strong adhesion through unique structural adaptations in diverse conditions, frog toe pads are particularly notable. Frogs exhibit effective wet adhesion due to mucus‐filled microstructures on their toe pads (top, **Figure** **2**a).^[^ ^45^ ^]^ These pads feature hexagonally arranged cells (diameter: 10–15 µm), and each contains hemispherical concave cavities that secrete mucus, forming a continuous fluid film at the adhesive interface that enhances adhesion through capillary connections and hydrodynamic interactions.^[^ ^53^, ^54^, ^55^ ^]^ A frog‐inspired, biodegradable adhesive film was developed using poly(glycerol sebacate) (PGS), with a glycerol‐coated hierarchical surface (bottom, Figure 2a). Developed frog‐inspired adhesive (FIA) films – a bioinspired microstructure composed of a hexagonal array and concave hemispherical cups (Figure 2b) – were evaluated for the shear adhesion performance, including porcine heart, lung, and liver (Figure 2c). Among the tested variants (e.g., flat, FIA, concave‐cup FIA (c‐FIA), and oil‐coated c‐FIA (o‐c‐FIA)), the o‐c‐FIA exhibited the highest shear adhesion, achieving up to 22.7/19.9/18.0 kPa on the heart/lung/liver, respectively. Such enhancement was attributed to synergistic effects between the glycerol‐based mucus mimic and the hierarchical microstructure, which amplified capillary and viscous forces at the tissue interface. Notably, adhesion strength was also influenced by the intrinsic moisture content of each tissue.^[^ ^56^ ^]^ Long‐term adhesion tests were conducted to assess the durability of adhesion during biodegradation, where ≈77% of the initial adhesion was retained even after ≈20% material degradation, which is consistent with the surface erosion behavior of PGS (Figure 2d).^[^ ^57^ ^]^ Cytocompatibility was confirmed via (3‐(4,5‐dimethylthiazol‐2‐yl)‐2,5‐diphenyltetrazolium bromide) (MTT) assay using Hep G2 liver cells,^[^ ^58^, ^59^ ^]^ showing ≈94% viability without detectable cytotoxicity (Figure 2e). On curved and hydrated organ surfaces, including porcine liver/lung/heart, the o‐c‐FIA film maintained stable and conformal contact, in contrast to the flat film as a control, which exhibited slippage (Figure 2f). In addition to frog‐inspired designs, gecko‐inspired biodegradable adhesives have been developed using poly(glycerol sebacate acrylate) (PGSA) with tunable crosslink density and biodegradability.^[^ ^60^ ^]^ Optimized nanopillar structures showed that adhesion strength strongly depended on tip‐to‐pitch and tip‐to‐base ratios, with lower values improving tissue conformity. Oxidized dextran functionalization further enhanced wet‐tissue adhesion, achieving over a two‐fold increase (from ≈0.3 to ≈0.7 N cm^−2^) after 48 h of implantation. PGSA with a degree of acrylation (DA) 0.3 exhibited ≈12% weight loss after one week, while DA 0.8 showed ≈5%, confirming slower degradation with higher crosslink density. Histological analysis revealed mild inflammation and no giant cell formation, underscoring the potential of nanostructured biodegradable polymers for robust and biocompatible adhesives.

In contrast to surface‐anchored microstructures that enhance adhesion through direct contact with tissues or substrates, another structural strategy involves particulate architectures at the interface to reinforce interfacial bonding. Schematic illustrations highlight the limitations of conventional mesoporous silica nanoparticles (MSNs) coatings, where excessive particle accumulation formed layers thicker than the hydrogel penetration depth and reduced adhesion (top, Figure 2g),^[^ ^22^, ^61^ ^]^ and introduced supraballs – cohesive spherical aggregates of MSNs – as an alternative structural design that enhanced interfacial adhesion by increasing contact area and interparticle cohesion (bottom, Figure 2g).^[^ ^16^ ^]^ A set of images shows the morphological evolution of silica nanoparticle aggregates produced via spray‐drying and thermal processing (Figure 2h).^[^ ^62^ ^]^ Spherical aggregates of nonporous silica nanoparticles (SB‐SNs), exhibit an average diameter of ≈3.5 µm with smooth surfaces and densely packed internal structures (top, Figure 2h). Supraballs of mesoporous silica nanoparticles (SB‐MSNs) were slightly larger (≈4 µm), and retained the porous architecture after thermal treatment (middle, Figure 2h). To introduce macroporosity, mesoporous nanoparticles were co‐spray‐dried with 200 nm polystyrene beads and subsequently heat‐treated to remove the sacrificial component. The resulting macroporous supraballs exhibit a comparable diameter (≈3.3 µm) with enlarged surface pores and increased pore volume (bottom, Figure 2h). To evaluate the interfacial reinforcement behavior of supraballs in hydrogel bonding, three types of supraballs, SB‐SNs, SB‐MSNs, and MSB‐MSNs, were positioned between two PDMA hydrogel substrates, respectively (Figure 2i). The resulting adhesion energy was plotted as a function of the total outer specific surface area (a_ETO_), showing that both supraballs and unaggregated nanoparticles exhibited similar adhesion behavior at low a_ETO_ values (<50), as most particles participated in interface bridging (Figure 2j). However, supraballs demonstrated significantly enhanced adhesion compared to unaggregated nanoparticles at higher a_ETO_ values. This increase was attributed to the larger size and cohesive structure, which prevented them from becoming fully embedded within a single hydrogel phase. Instead, they predominantly remained at the interface, effectively connecting the two hydrogel networks and enhancing interfacial bonding. Furthermore, biodegradation behavior of supraballs was assessed in simulated body fluid (SBF) by measuring the release of silicate ions over time using inductively coupled plasma–optical emission spectrometry (ICP–OES) (Figure 2k). All particle types exhibited comparable degradation kinetics, with ≈90% mass loss observed within 24 h. To examine the effect of degradation on adhesion, the particles were pre‐immersed in SBF for varying durations, then redispersed and applied to hydrogels under identical conditions (Figure 2l). Short‐term immersion (<1 h) led to a noticeable increase in adhesion for all samples, particularly for supraballs, likely due to surface roughening and partial fragmentation. While longer immersion, adhesion gradually decreased.

Altogether, these structurally engineered bioadhesive platforms, from surface‐bound microtopographies to embedded particulate systems, highlight the growing potential of geometry‐driven strategies in overcoming persistent challenges in wet adhesion, tissue conformity, and biodegradability. Such approaches hold promise not only for sutureless tissue repair, but also for broader applications, including regenerative medicine, implantable devices, and dynamic soft‐tissue integration.

## 3 Chemical Approaches

### 3.1 Hydrogen Bonding

**Figure 3:**
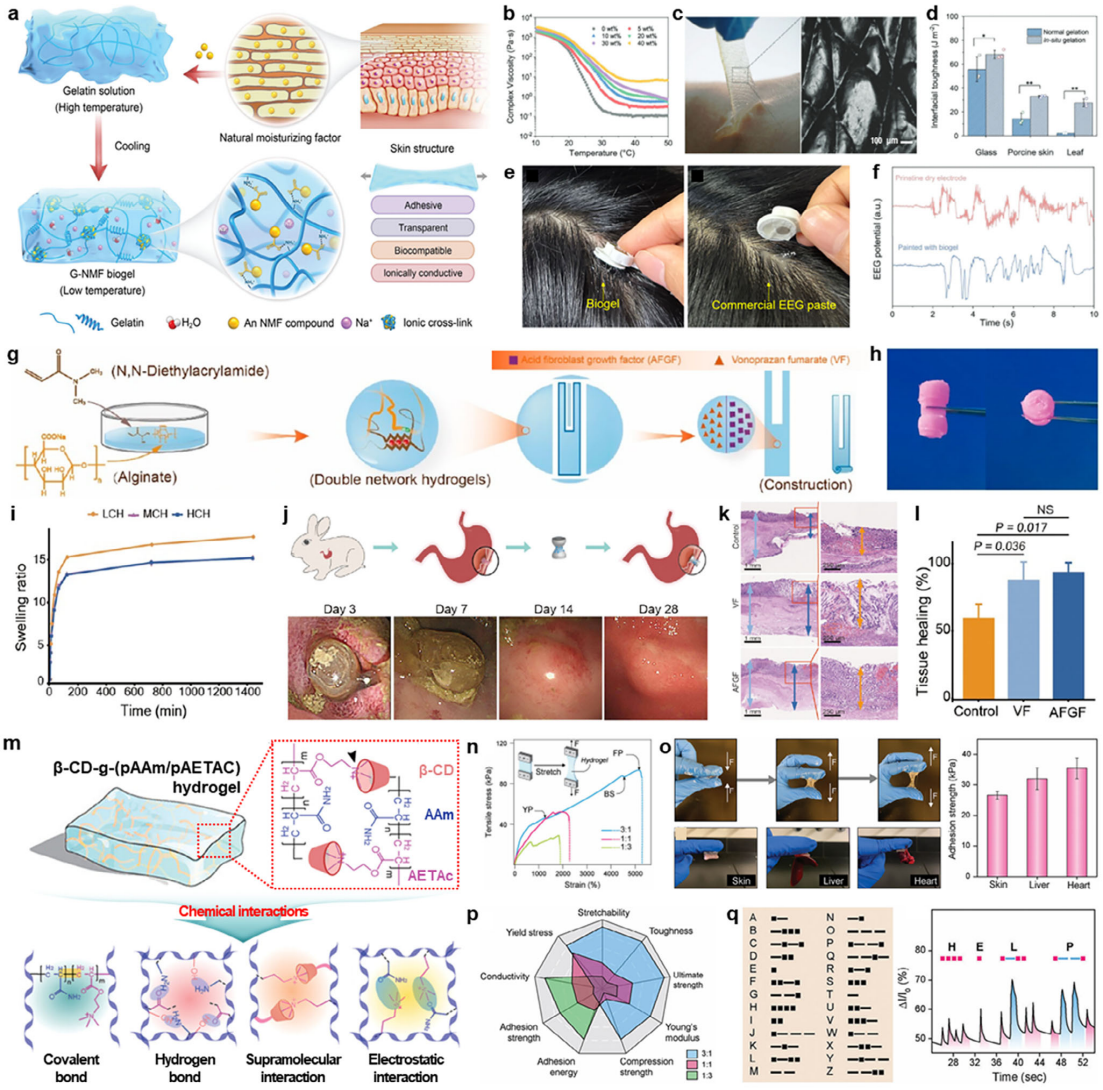
Hydrogen bonding. a) Schematic illustration of a biocompatible, transparent, ionically conductive adhesive biogel (G‐NMF biogel), synthesized by ionic crosslinking between gelatin and a natural moisturizing factor (NMF), sodium pyrrolidone carboxylic acid (PCA‐Na). b) Complex viscosities of biogels with different NMF concentrations of 0, 5, 10, 20, 30, and 40 wt.%. c) Photograph showing the detachment of the biogel from the skin (left) and a magnified optical image of the biogel surface replicating a fingerprint pattern (right). d) Interfacial toughness of biogels is either attached after gelation (normal gelation) or directly formed on substrates (in situ gelation), including glass, porcine skin, and leaf surfaces. e) Photographs of biosignal sensing probes attached to hairy scalp regions using G‐NMF biogel (left) and a commercial electroencephalography (EEG) paste (right). f) Comparison of EEG signals recorded with dry electrodes during eye blinking, with and without the G‐NMF biogel as an interfacial layer. Reproduced with permission.^[^ ^31^ ^]^ Copyright 2024, Wiley. g) Schematic illustration of the fabrication process for a mushroom‐cap‐inspired hyperboloid device: i) synthesis of acid‐resistant, tough, and elastic double‐network hydrogels via crosslinking of dimethylacrylamide (DMA), N,N’‐cystaminebis(acrylamide) (CBA), and sodium alginate (SA), with the addition of acidic fibroblast growth factor (AFGF) and vonoprazan fumarate (VF), ii) formation of a thin hydrogel sheet, followed by cutting into the desired shape, and iii) rolling the sheet into a hyperboloid cap‐stick structure. h) Photographs of the fabricated device in side (left) and top (right) views. i) Swelling ratios of hydrogels synthesized with different CBA amounts in simulated gastric fluid: 0.04 wt.% (LCH), 0.15 wt.% (MCH), and 0.3 wt.% (HCH). j) Schematic of the surgical procedure for treating acute gastric perforation in a rabbit using the hyperboloid hydrogel device (top) and gastroscopic images of the treated perforation at 3, 7, 14, and 28 days post‐operation (bottom). k) H&E‐stained histological images of gastric walls treated with an unloaded hydrogel device (Control), VF‐loaded hydrogel device (VF), and AFGF‐loaded hydrogel device (AFGF), where light blue, blue, and orange arrows indicated intact and healed walls, and the villi, respectively. l) Comparative analysis of gastric wall healing performance among the Control, VF, and AFGF groups. Reproduced with permission.^[^ ^27^ ^]^ Copyright 2023, American Chemical Society. m) Synthesis of a highly stretchable, conductive, and transparent bioadhesive hydrogel via crosslinking 𝛽‐cyclodextrin (𝛽‐CD) and polyacrylamide (pAAm) with a bio ionic liquid, 2‐(acryloyloxy)ethyltrimethylammonium chloride (pAETAc). n) Stress–strain curves of bioadhesive hydrogels with different AAm:AETAc ratios: 3:1, 1:1, and 1:3. o) Optical images showing the adhesion of the hydrogel (1:1) to gloves (top left) and biological tissues, including skin, liver, and heart (bottom left), and measured lab‐shear adhesion forces between the hydrogel and the respective tissues (right). p) Comparison of mechanical, electrical, and adhesive properties of bioadhesive hydrogels with AAm:AETAc ratios of 3:1, 1:1, and 1:3. q) International Morse code chart (left) and demonstration of the hydrogel (1:1) as a real‐time human motion sensor for detecting the word “HELP” using Morse code signals. Reproduced with permission.^[^ ^30^ ^]^ Copyright 2024, Wiley.

Hydrogen bonding represents a highly effective approach for bioadhesives due to its reversible and dynamic characteristics. Such interactions enable not only strong initial adhesion, but also stress dissipation under mechanical deformation. **Figure** **3**a illustrates a biocompatible, transparent, and ionically conductive adhesive biogel (G‐NMF biogel), synthesized via ionic crosslinking between amino groups of gelatin and carboxyl groups of a natural moisturizing factor (NMF), sodium pyrrolidone carboxylic acid (PCA‐Na).^[^ ^31^ ^]^ Compared to conventional gelatin hydrogels, this biogel exhibited substantially enhanced mechanical robustness due to the ionic crosslink, improved ionic conductivity from mobile sodium ions, and enhanced water retention capabilities via strong hydrogen bonding with water. Rheological characterization in Figure 3b confirmed that the complex viscosity proportionally increased with PCA‐Na concentrations, which was correlated with improved mechanical stability arising from increased crosslink density. Notably, the gel‐to‐liquid phase transition observed between 25 and 35 °C—marked by a dramatic drop in viscosity—enabled the biogel to form intimate contact with biological substrates, as shown in Figure 3c where the biogel effectively replicated fine surface textures such as fingerprint patterns upon detachment from the skin. When directly applied via in situ gelation, the biogel achieved significantly higher interfacial toughness on various substrates, especially porcine skin and plant leaves, than pre‐gelled samples (Figure 3d), owing to effective penetration of the fluidic biogel into microscale surface irregularities before solidification. The practical applicability of the G‐NMF biogel in biomedical applications was validated as an interfacial adhesive for biosignal sensing. When applied to hairy scalp regions, it exhibited strong adhesion compared to commercial electroencephalography (EEG) pastes (Figure 3e). Therefore, measured EEG signals using dry electrodes with the biogel as an interfacial layer exhibited reduced noise and enhanced signal fidelity during dynamic movements such as eye blinking, compared to electrodes without the biogel (Figure 3f), highlighting the potential for robust, reliable, and biocompatible interfaces for electrophysiological recording.

Hydrogen bonding‐based adhesives are particularly effective in wet environments. As a representative example, Figure 3g–l depict a mushroom‐cap‐inspired hyperboloid bioadhesive hydrogel device designed for gastric perforations under harsh acidic conditions.^[^ ^27^ ^]^ The fabrication process (Figure 3g) involved synthesizing a tough and elastic double‐network hydrogel through the crosslinking of *N,N*‐dimethylacrylamide (DMA), sodium alginate (SA), and a biodegradable crosslinker, *N,N′*‐cystaminebis(acrylamide) (CBA), and incorporating acidic fibroblast growth factor (AFGF) and vonoprazan fumarate (VF) to promote healing and regulate intragastric pH level (Figure 3g). The hydrogel was then shaped into a thin hydrogel sheet, cut, and rolled into a hyperboloid structure capable of endoscopic deployment and swelling‐induced expansion upon reaching the target site (Figure 3h). Here, the alginate‐based matrix, rich in carboxyl (–COOH) and hydroxyl (–OH) groups, enabled the formation of multiple hydrogen bonds with complementary functional groups, including amine (–NH_2_), hydroxyl, and carbonyl moieties. These reversible, non‐covalent interactions facilitated conformal interfacial contact and robust adhesion, even in the presence of gastric fluids that typically disrupt physical bonding. In acidic conditions, protonation of carboxylate groups further stabilized hydrogen bonding, thereby enhancing adhesion and limiting premature hydrogel swelling. Figure 3i shows the tunable swelling behaviors based on CBA concentration (LCH, 0.04%; MCH, 0.15%; HCH, 0.3%), which influenced the crosslink density. Low CBA contents allowed large swelling ratios while maintaining sufficient structural integrity, ensuring reliable expansion and sealing under acidic conditions. The clinical relevance of this hydrogen bonding‐mediated adhesion strategy was demonstrated in Figure 3j. When applied in a rabbit model of acute gastric perforation, the device achieved stable adhesion and maintained effective sealing over 28 days post‐operation, as evidenced by gastroscopic monitoring. Moreover, integrating additional functionalities, such as magnetic actuation,^[^ ^63^ ^]^ catheter‐assisted deployment,^[^ ^64^ ^]^ and real‐time imaging guidance (e.g., X‐ray or computed tomography (CT)), could further enable non‐surgical, site‐specific positioning and robust adhesion of hydrogel‐based adhesives, highlighting their potential for advanced biomedical applications. Histological analysis revealed enhanced mucosal regeneration and reduced inflammation in the VF and AFGF‐loaded groups compared to the control (Figure 3k). Quantitative assessments further confirmed superior tissue healing performance in the drug‐loaded devices (Figure 3l), highlighting the promise for minimally invasive, soft tissue‐targeted biomedical applications.

Hydrogen bonding serves not only as a key element for wet‐tissue adhesion but also as a tunable interaction that contributes to the mechanical integrity, ionic conductivity, and dynamic sensing performance of biodegradable hydrogels. A. Roy et al. engineered a highly stretchable, conductive, and transparent bioadhesive hydrogel for use in flexible sensors (Figure 3m).^[^ ^30^ ^]^ This system leveraged multiple hydrogen bonding interactions – between the amide groups of polyacrylamide (pAAm) and hydroxyl groups of β‐cyclodextrin (β‐CD), as well as between β‐CD and water molecules – yielding a physically crosslinked network with excellent mechanical resilience and conformability. Incorporation of a bio‐ionic liquid monomer, 2‐(acryloyloxy)ethyltrimethylammonium chloride (AETAc), enhanced ionic conductivity and introduced electrostatic interactions with surrounding polar groups, thereby reinforcing the gel matrix while preserving optical transparency and flexibility. Accordingly, increasing the ionic liquid content relative to AAm led to a higher mechanical modulus but reduced stretchability (Figure 3n). Additionally, the hydrogel demonstrated strong wet adhesion (> ≈30 kPa) to diverse biological tissues including skin, liver, and heart (Figure 3o), primarily through hydrogen bonding between gel‐phase hydroxyl/amide groups and tissue‐surface amines and carboxylic acids. Comparative evaluation across different formulations (3:1, 1:1, and 1:3 AAm:AETAc ratios) confirmed the synergistic contribution of hydrogen bonding and ionic components to the hydrogel's physical, mechanical, and electrical properties (Figure 3p). The 1:1 ratio was found to be optimal, which provided a well‐balanced network density, ionic mobility, and adhesive strength. Figure 3q highlights such multifunctionality in real‐time biomechanical sensing. The hydrogel with a 1:1 AAm:AETAc ratio functioned as a handwriting detection sensor, generating distinct electrical signals in response to static (3 s) and dynamic (1 s) touches, which correspond to “dots” and dashes″. This feature enabled reliable motion‐based communication, e.g., encoding the word “HELP” in International Morse code, underscoring the potential as skin‐integrated biosensors for wearable electronics, emergency signaling, and human–machine interfaces, with the added benefit of biocompatibility and biodegradability.

### 3.2 Catechol‐Based Adhesion

Dopamine, inspired by the adhesive proteins secreted by mussels, has been widely employed as a key component in the development of chemically engineered bioadhesives. The catechol groups in dopamine exhibit strong affinity toward various surfaces through hydrogen bonding, *π*–*π* interactions, and covalent crosslinking under oxidative conditions, enabling robust adhesion even under wet or dynamic environments. Building on these properties, recent efforts have focused on incorporating dopamine chemistry into biodegradable adhesive systems for biomedical use.

**Figure 4:**
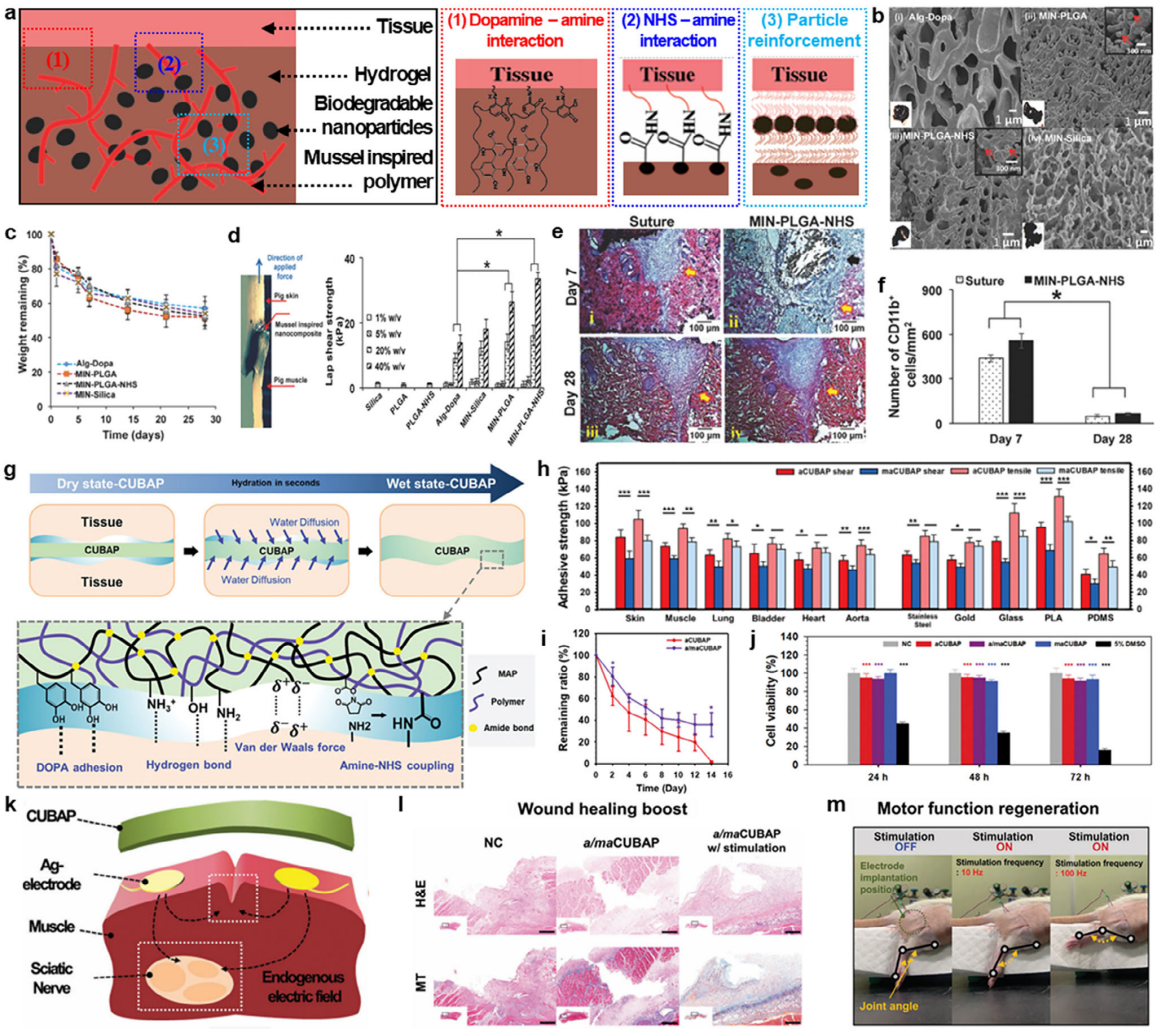
Catechol‐based adhesion. a) Schematic illustration of mussel‐inspired nanocomposite (MIN) hydrogels prepared by grafting dopamine (Dopa) onto alginate (Alg) in PBS and blending with biodegradable poly(lactide‐co‐glycolide) (PLGA) or silica nanoparticles (top) and the tissue adhesion mechanisms (bottom), involving three distinct interactions: 1) covalent bonding between oxidized dopamine and amine groups in tissues, 2) physical adsorption of nanoparticles at hydrogel‐tissue interfaces, and 3) coupling via N‐hydroxysuccinimide (NHS) groups on nanoparticles with tissue amine groups. b) SEM images of surface morphologies of various MINs: i) Alg‐Dopa, ii) MIN‐PLGA, iii) MIN‐PLGA‐NHS, and iv) MIN‐Silica, with insets showing a magnified view of embedded nanoparticles (top right) and the optical images (bottom left). c) Weight changes of MINs immersed in PBS at 37  °C over 28 days. d) Measured lap shear strengths of silica, PLGA, PLGA‐NHS, and MINs with Alg‐Dopa polymer concentrations of 1, 5, 20, and 40 w/v% (right). e) H&E‐stained histological images of wound sites closed with either sutures or MIN‐PLGA‐NHS at 7 and 28 days post‐surgery. f) Quantitative analysis of CD11b⁺ cell densities (cells/mm^2^) near wound sites treated with sutures or MIN–PLGA–NHS. Reproduced with permission.^[^ ^21^ ^]^ Copyright 2018, Wiley‐VCH. g) Schematic of the tissue adhesion mechanism of customized underwater bioadhesive patches (CUBAPs), where rapid hydration enabled conformal contact with tissue surfaces and strong bonding via Dopa‐mediated interaction, hydrogen bonding, van der Waals force, and NHS‐amine coupling. h) Wet adhesion strengths of various CUBAP types on biological tissues and non‐biological substrates; aCUBAP, maCUBAP, and a/maCUBAP refer to formulations using muscle adhesive protein (MAP)‐acryloyl/acrylic acid, MAP‐methacryloyl/methacrylic acid, and their 1:1 mixture, respectively; PLA and PDMS are poly(lactic acid) and polydimethylsiloxane. i) Weight retention ratios of aCUBAP and a/maCUBAP during 15‐day subcutaneous implantation in mice. j) Cytocompatibility of CUBAPs assessed by NIH3T3 cell cultures for 24, 48, and 72 h. k) Schematic depicting CUBAP application on silver electrodes‐implanted muscle tissues for wound healing and motor function regeneration. l) H&E and Masson's trichrome‐stained histological images of injured muscle tissues in negative control (NC) and a/maCUBAP‐applied groups, with or without electrical stimulation (600 µA, 0.8 Hz). m) Photographs of hind limb movements induced by electrical stimulation (100 µA, 10 or 100 Hz) via gold electrodes implanted over the biceps femoris muscle (green dashed circle). Reproduced with permission.^[^ ^14^ ^]^ Copyright 2023, Wiley‐VCH.

**Figure** **4**a illustrates strategies of mussel‐inspired tissue adhesive that synergistically integrate multiple adhesion mechanisms to achieve strong bonding under wet conditions.^[^ ^21^ ^]^ The key features of this system include catechol‐mediated chemistry, nanoparticles bridging, and covalent bonding via N‐hydroxysuccinimide (NHS) group. First, dopamine‐functionalized alginate introduced catechol groups that can play a central role in adhesion by diverse interactions such as hydrogen bonding, *π*–*π* stacking, and coordination with metal ions in biological environments. Additionally, catechols were converted into reactive quinones that can form covalent bonds with nucleophilic groups (e.g., amines or thiols on tissue), ensuring stable interfacial anchoring. Second, biodegradable poly(lactide‐co‐glycolide) nanoparticles (PLGA NPs) further enhanced adhesion by physically bridging the hydrogel and tissue interfaces due to their small size and high aspect ratio. Third, strengthening the interface, NHS grafted onto the PLGA NPs facilitated covalent reactions with amines on tissues. All of these multi‐modal strategies were combined with physical reinforcement, resulting in improved performance under biological wet conditions. Mussel‐inspired nanocomposites (MINs) were prepared by blending dopamine‐mediated alginate hydrogel (Alg‐Dopa) with PLGA (MIN‐PLGA), PLGA‐NHS (MIN‐PLGA‐NHS), and silica nanoparticles (MIN‐Silica), and the resulting composites exhibited a dark brown color with porous structures (Figure 4b). These MINs exhibited gradual hydrolytic degradation in phosphate‐buffered saline (PBS) at 37 °C, reaching 53 ± 1% degradation after 4 weeks (Figure 4c). This result indicates that the incorporation of PLGA nanoparticles slightly accelerated the overall degradation of the MIN compared to Alg‐Dopa. To assess the adhesion performance, MINs were tested at porcine skin‐muscle interfaces and achieved a lap shear strength (33 ± 3 kPa), more than twice that of Alg‐Dopa (Figure 4d). This is because the nanoparticles were anchored to the surfaces and dissipated energy across the interfaces, limiting crack propagation.^[^ ^65^ ^]^ Toxicity investigation through dorsal skin wounds of Spraque–Dawley rats was performed, and the wounds were closed by suture and MIN‐PLGA‐NHS, respectively. Hematoxylin and eosin (H&E) staining and immunohistochemistry were performed at weeks 1 and 4 to evaluate foreign body reactions and immune responses. In the MIN‐PLGA‐NHS group, H&E‐stained images showed cell infiltration and material degradation at week 1, with complete adhesive resorption and wound closure by week 4 (Figure 4e). CD11b⁺ inflammatory cells decreased similarly in both groups over time, indicating comparable resolution of inflammation (Figure 4f).

To further explore catechol‐mediated adhesion in a distinct formulation, a nanoparticle‐free, film‐based platform was developed to enable broader tissue compatibility and functional integration. Figure 4g depicts the working principle of a customizable underwater bioadhesive patch (CUBAP),^[^ ^14^ ^]^ which relies on a water‐triggered increase in surface energy, inducing DOPA‐mediated adhesion, hydrogen bonding, van der Waals interactions, and amine‐NHS coupling. These CUBAPs were prepared via mixing mussel adhesive proteins (MAP)‐(meth)acryloyl, (meth)acrylic acid, and photoinitiator, followed by UV crosslinking, resulting in a poly((meta)acrylic acid)‐based network in three formulations: CUBAP with MAP‐acryloyl/acrylic acid (aCUBAP), CUBAP with MAP‐(meth)acryloyl/(meth)acryic acid (maCUBAP), and a 1:1 mixture of aCUBAP and maCUBAP (a/maCUBAP). Multifunctional adhesion capability of CUBAP was demonstrated by its strong wet adhesion to various internal organs, with the highest shear and tensile strengths observed on skin (83.6 and 104.3 kPa) and muscle (73.0 and 93.8 kPa), and comparable performance on lung, bladder, heart, and aorta (Figure 4h). Also, the double‐sided adhesion enabled binding to non‐biological surfaces, including poly(lactic acid) (PLA), glass, metal, and polydimethylsiloxane (PDMS), with the strongest adhesion found on PLA. To evaluate in vivo biodegradation, aCUBAP and a/maCUBAP were subcutaneously implanted into the dorsal region of C57BL/6 mice for 15 days (Figure 4i). aCUBAP exhibited a consistently faster degradation rate than a/maCUBAP, with only 1.6% remaining compared to 36% for a/maCUBAP at day 15, indicating that a higher (meth)acrylic acid content reduced the degradation rate. Cytocompatibility was confirmed via cell counting kit‐8 (CCK‐8) assay using HaCaT human keratinocyte cells after 72 h of culture, showing over 94% viability without detectable cytotoxicity (Figure 4j). Based on the overall properties, the CUBAP system was applied in a dual‐functional electric patch for wound healing and neurorehabilitation (Figure 4k). a/maCUBAP was placed on a muscle incision and provided electrical stimulation (600 µA, 0.8 Hz, 3 min, days 2/4/6), leading to enhanced tissue regeneration as confirmed by H&E and Masson's trichrome (MT) staining (Figure 4l). For neuromodulation, the patch was positioned near the sciatic nerve, inducing frequency‐dependent limb movements with joint angles of 173 and 98° at 100 and 10 Hz, respectively (Figure 4m).

These findings highlight the potential of dopamine‐mediated adhesives as adaptable platforms for biomedical applications, offering tunable properties and functional integration.

### 3.3 Amine‐Carboxyl Interactions

**Figure 5:**
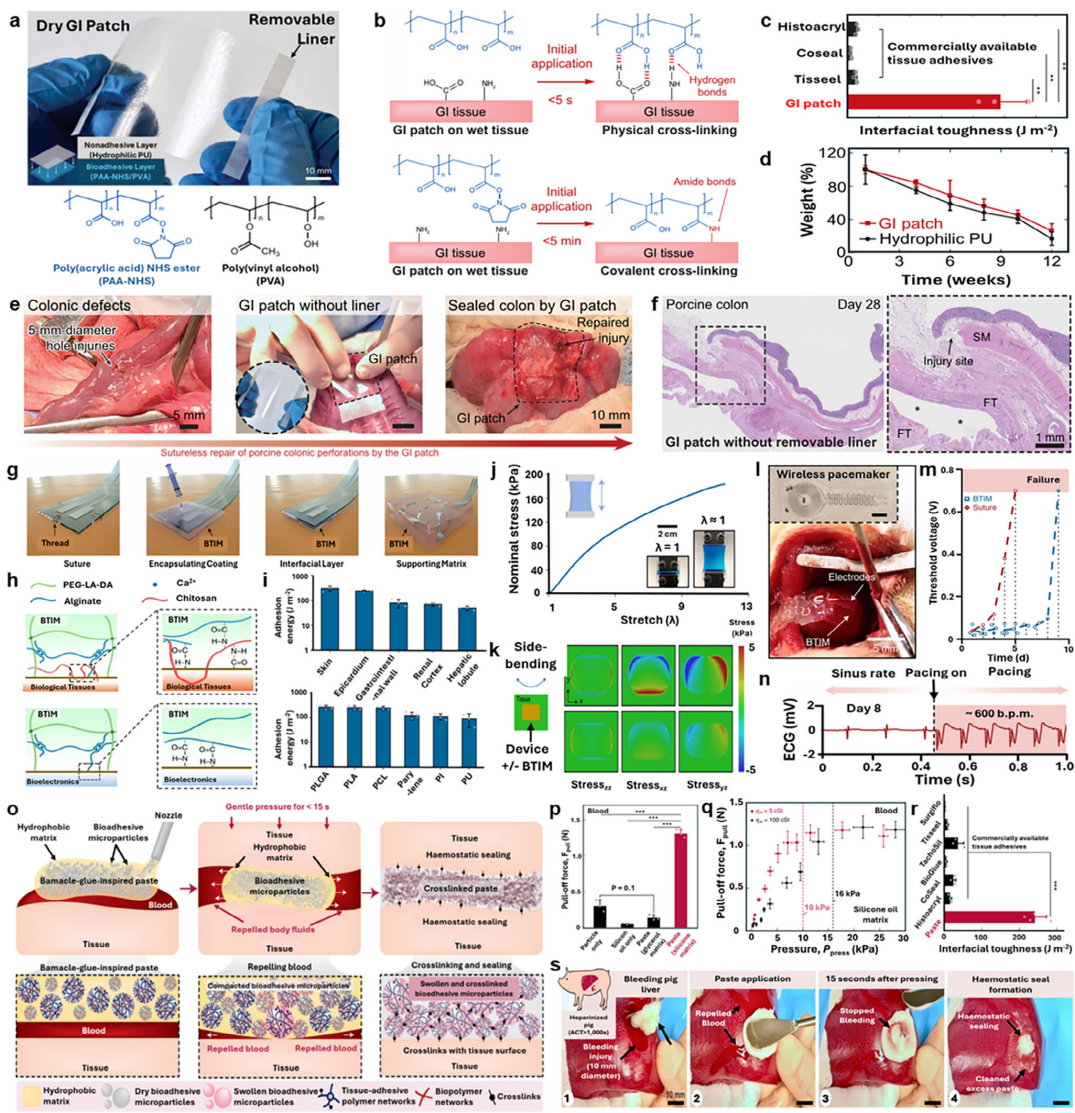
Amine‐carboxyl interactions. a) Photograph and schematic illustration (inset) of a gastrointestinal (GI) patch consisting of a nonadhesive top layer made of hydrophilic polyurethane (PU)) and a bioadhesive bottom layer composed of poly(acrylic acid) N‐hydroxysuccinimide (NHS) ester (PAA‐NHS) and poly(vinyl alcohol) (PVA) (top), with chemical structures of the adhesive components (bottom). b) Schematic showing the rapid wet tissue adhesion mechanism of the GI patch, initially mediated by physical cross‐linking via hydrogen bonding (top), followed by covalent cross‐linking through amide bond formation between carboxyl groups in the adhesive and amine groups on tissue surfaces. c) Comparison of interfacial toughness of the GI patch against commercially available tissue adhesives using ex vivo porcine colon tissue. d) In vivo degradation profiles of the GI patch and hydrophilic PU during subcutaneous implantation in rats. e) Demonstration of sutureless repair of porcine colonic perforations with the GI patch: defect generation using a biopsy punch (left), patch application on defects (middle), and complete sealing of defects (right). f) H&E‐stained histological images of repaired porcine colon defects 28 days post‐treatment, where SM and FT denote smooth muscle and fibrotic tissue, respectively. Reproduced with permission.^[^ ^15^ ^]^ Copyright 2022, AAAS. g) Applications of a bioelectronic‐tissue interface material (BTIM) composed of polyethylene glycol‐lactide‐diacrylate (PEG‐LA‐DA), alginate, chitosan, and calcium ions, functioning as encapsulating coatings, interfacial layers, and supporting matrices to enhance integration of bioelectronics with tissues compared to traditional sutures. h) Schematic illustrations of interfacial bonding mechanisms between BTIM to biological tissues or bioelectronic devices via amide bond formation. i) Measured adhesion energies of BTIM on various biological tissues and bioelectronic device materials. j) Stress–strain curve of a BTIM film, with the optical images before and after elongation (insets). k) Finite‐element analysis of shear and normal stress distributions at the bioelectronics‐tissue interface with and without BTIM interfacial layer. l) Demonstration of securing a wireless cardiac pacemaker in a rat model using BTIM as the encapsulating coating, with an optical image of the device featuring a radiofrequency power harvester and pacing electrodes in the inset. m) Measurement of pacing threshold voltages for pacemakers fixed with BTIM and sutures over time, indicating prolonged functional stability with BTIM. n) Representative electrocardiography (ECG) recordings on day 8 post‐implantation, illustrating cardiac responses before and after pacing with the BTIM‐assisted pacemaker. Reproduced with permission.^[^ ^35^ ^]^ Copyright 2021, Nature Publishing Group. o) Schematics of tissue adhesion mechanism of a barnacle‐glue‐inspired paste, composed of bioadhesive microparticles embedded in a hydrophobic silicone oil matrix, where the oil repels blood at the paste‐tissue interface, facilitating cross‐linking between carboxyl groups on the microparticles and tissue amine groups to achieve strong adhesion. p) Pull‐off force measurements of tissues sealed with different bioadhesive formulations in a heparinized porcine blood bath. q) Pull‐off forces of tissues sealed with pastes with different viscosities of silicone oil matrix (5 and 100 cSt) as a function of applied pressure, where dashed lines indicate threshold pressures. r) Comparison of interfacial toughness between the barnacle‐glue‐inspired paste and commercial adhesives on blood‐covered porcine skin. s) Photographs demonstrating in vivo haemostatic sealing of a bleeding liver injury in an anticoagulated pig using the barnacle‐glue‐inspired paste. Reproduced with permission.^[^ ^17^ ^]^ Copyright 2021, Nature Publishing Group.

Despite improvements in tissue adhesives and surgical techniques, anastomotic leaks after gastrointestinal (GI) surgery continue to be a serious complication with high morbidity and mortality. To overcome these drawbacks, Wu et al. created a commercially available GI bioadhesive patch that uses the complementary action of carboxyl (‐COOH) and amine (‐NH_2_) groups to seal defects in a quick, robust, and sutureless fashion.^[^ ^15^ ^]^ **Figure** **5**a presents a patch combining a hydrophilic polyurethane (PU) nonadhesive (top layer) with a dry adhesive (bottom layer) made of poly(acrylic acid)‐N‐hydroxysuccinimide ester (PAA‐NHS) and poly(vinyl alcohol) (PVA). Using adhesion mechanisms established in previous works,^[^ ^2^, ^49^ ^]^ interfacial water absorption upon application to wet tissue initiated hydrogen bonding and formed amide bonds at the tissue interface, providing strong covalent cross‐linking (Figure 5b). These synergistic chemical reactions enabled better adhesive performance in terms of interfacial toughness, tensile strength, and shear strength than commercial sealants such as Histoacryl, Coseal, and Tisseel (Figure 5c). Such outstanding performance was achieved without the need for heat, UV curing, or external stimuli—a major drawback of many adhesives currently on the market. Figure 5d illustrates the dissolution behavior of the patches implanted in a rat model, revealing gradual weight loss over 12 weeks due to hydrolysis of ester and amide bonds. As a biomedical application, Figure 5e shows effective sutureless repair of GI perforation in a porcine model. The patch allowed for facile and atraumatic repair of injuries in less than 10 s with minimal inflammation, unlike sutures that frequently cause needle damage and fibrosis (Figure 5f).

Figure 5g illustrates a biodegradable, photocurable adhesive that offers strong integration at the interface between flexible electronics and soft tissues, referred to as a bioadhesive‐based tissue–interface mechanism (BTIM).^[^ ^35^ ^]^ The BTIM employed a dual‐component adhesive system consisting of sodium alginate (SA) and PEG–lactide‐diacrylate (PEG‐LA‐DA), with chitosan or EDC/NHS added to improve covalent coupling. The system provided three interface strategies that enable conformal and stable bonding across a variety of tissues: encapsulation, interfacial adhesion, and matrix reinforcement. Through interactions between amine and carboxyl groups, covalent cross‐linking created strong amide bonds that outperformed pressure‐sensitive adhesives and hydrogels when applied to tissues and device surfaces (Figure 5h,i). High elasticity and effective stress dissipation in dynamic biological environments were attributed to mechanical resilience, which was confirmed by stress–strain analysis (Figure 5j) and finite‐element modeling (Figure 5k). The BTIM was also applied to a wireless cardiac pacemaker system attached to the epicardium via an encapsulation interface, validating the utility in practical application (Figure 5l). Such a strategy allowed for suture‐free, trauma‐minimized fixation, thus eliminating suture‐related issues such as fibrosis and pacing failure. In contrast to the suture process that displayed early malfunction, electrical measurements verified steady pacemaker function, with the steady threshold voltage over time (Figure 5m). Sustained functionality was confirmed by ECG recordings on days 1, 4, and 8. The ECG recordings from day 8 after implantation are displayed in Figure 5n, which also shows the cardiac responses both before and after pacing with the BTIM‐assisted pacemaker. Suitable for wearable, implantable, or temporary devices in neural, gastrointestinal, and cardiovascular applications, this platform extended the potential of medical adhesives to next‐generation, multifunctional systems.

A biodegradable adhesive paste inspired by barnacles was created for coagulopathic, high‐bleed surgical settings.^[^ ^17^ ^]^ The adhesive blended bioadhesive microparticles with a hydrophobic silicone oil matrix to mimic barnacle adhesion, which used a two‐phase secretion to displace contaminants and to obtain rapid fixation (Figure 5o). Blood was repelled by the oil under pressure, and the microparticles aggregated together to form a dense matrix, creating amid and hydrogen bonds between the carboxyl and amine groups. This allowed for quick (less than 15 s) and stimuli‐free sealing, which would be useful for trauma surgery where conventional hemostats did not work properly. According to quantitative performance tests, sealing strength rose as oil viscosity and applied pressure increased (Figure 5p,q), which is consistent with granular suspension theory predictions. It showed remarkable wet adhesion and outperformed commercial sealants on porcine tissue covered in blood (Figure 5r). In vivo tests revealed minimal inflammation, macrophage recruitment, and progressive breakdown (Figure 5s), and its clinical safety profile was further supported by degradation through the hydrolysis of ester and amide bonds. This system provides trauma‐ready, next‐generation hemostasis with strong wet adhesion, biodegradability, and the ability to be integrated into electronics and surgical procedures.

Amine‐carboxyl interaction‐based adhesives are strong adhesives with distinct biodegradation pathways. Under physiological aqueous conditions, amide bonds formed by amine–carboxyl condensation undergo enzymatic cleavage by amidases and proteases such as collagenase and cathepsins, as well as slow hydrolysis (rate constant typically 10^−7^–10^−6^ s^−1^ at 37 °C).^[^ ^15^ ^]^ Degradation is further accelerated by the oxidative pathways in inflamed tissues. Degradation timescales vary depending on the polymer composition: networks derived from chitosan or gelatin degrade more quickly (days to weeks), whereas adhesives based on poly (aspartic acid) lose ≈40–60% of their mass in 2–6 weeks in PBS (pH 7.4).^[^ ^66^ ^]^ Degradation also affects adhesion performance; for example, gelatin‐carbodiimide adhesives lost approximately half of their bonding strength after 14 days in PBS, whereas poly(aspartic acid) hydrogels lost all their adhesion after 30 days in vivo.^[^ ^67^ ^]^ Most of the degradation byproducts are short‐chain polymers (aspartates), oligopeptides, and amino acids. These compounds undergo natural metabolism, are water‐soluble, and are non‐toxic.^[^ ^35^ ^]^ The findings from tough hydrogel bioadhesives used in wound sealing and hemostasis,^[^ ^68^ ^]^ Janus bioadhesives for abdominal repair,^[^ ^69^ ^]^ and recent reviews of amine–carboxyl adhesives^[^ ^66^ ^]^ are agreeing with in vivo studies that further confirm degradation products such as chitooligosaccharides, poly(aspartates), and peptide fragments do not accumulate in organs and do not cause systemic toxicity or inflammatory responses.

When evaluating amine–carboxyl interaction‐based adhesives, biomechanical safety thresholds must be considered in addition to chemical stability and biodegradation behavior. Different tissues tolerate different maximum adhesion stresses before mechanical damage occurs. For example, skin can typically withstand interfacial shear stresses of ≈20–40 kPa before risking epidermal–dermal separation, while fragile tissues such as the gastrointestinal tract or dura mater often fail at much lower thresholds (≈5–15 kPa).^[^ ^17^ ^]^ Pericardial tissues and blood vessels can typically withstand ≈10–20 kPa.^[^ ^17^ ^]^ Although strong sealing is necessary, these ranges highlight that too much adhesion can raise the possibility of tearing, necrosis, or painful detachment. Importantly, the crosslinking density and degradation rate of amine–carboxyl adhesives can be modified, allowing formulations to be designed that stay within safe thresholds for each target organ.^[^ ^15^, ^17^ ^]^ This emphasizes the significance of tissue‐specific optimization.

### 3.4 Other Chemistries: Diazirine and Urea Linkages

**Figure 6:**
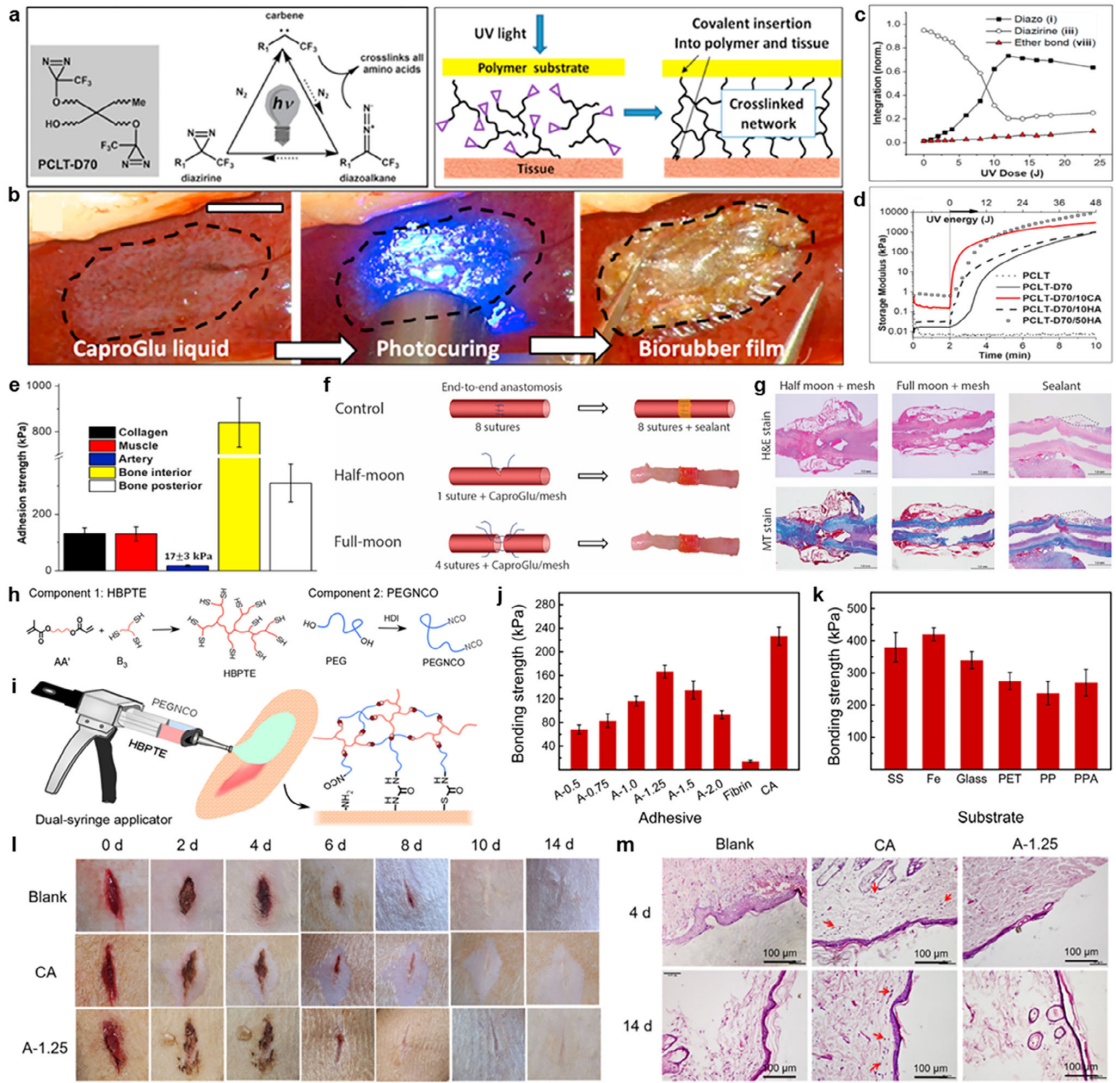
Carbene insertion and urea linkage. a) Synthetic pathway of CaproGlu via grafting UVA‐activated diazirine groups onto polycaprolactone triol (PCLT) (left), and the adhesion mechanism via carbene insertion with synthetic materials and tissues (right). b) Optical images showing the tissue adhesion process of CaproGlu on a hydrated rabbit liver surface. c) Curing kinetics of CaproGlu with PCLT/diazirine molar ratio of 1/2 (PCLT‐D70), highlighting diazo formation, diazirine depletion, and ether bond formation under UV irradiation. d) Photorheometry spectra of neat PCLT, PCLT‐D70, and PCLT‐D70 with 10 wt.% citric acid or hydroxyapatite, and with 50 wt.% hydroxyapatite (PCLT‐D70/10CA, PCLT‐D70/10HA, and PCLT‐D70/50HA, respectively), showing increased storage moduli upon UV‐induced crosslinking, further enhanced by additive incorporation. e) Lap shear adhesion strengths of PCLT‐D70 on hydrated collagen, PCLT‐D70/10CA on muscle tissues, and PCLT‐D70/50HA on bone tissues. f) Illustrations of CaproGlu‐assisted vascular anastomosis through mesh‐reinforced sealing for effective iliac artery repair in a rabbit model. g) Corresponding H&E and Masson's trichrome‐stained histological images of repaired arteries. Reproduced with permission.^[^ ^29^ ^]^ Copyright 2020, Elsevier. h) Synthesis of a dual‐component adhesive consisting of a thiol‐end capped hyperbranched polymer (HBPTE) and a hexamethylene diisocyanate‐capped polyethylene glycol (PEGNCO), where AA’, B3, and HDI represent 2‐(acryloyloxy)ethyl methacrylate, trimethylolpropane tris(3‐mercaptopropionate), and hexamethylene diisocyanate, respectively. i) Schematic illustrations of adhesive application using a dual‐syringe applicator and the tissue bonding mechanism involving interfacial interactions of free isocyanate (─NCO) groups on PEGNCO with thiol (─SH) groups on HBPTE (cohesion), as well as with primary amine (─NH_2_) and thiol groups on tissue surfaces (adhesion). j) Comparison of bonding strengths of adhesives with varying molar ratios of isocyanate to thiol groups (0.5, 0.75, 1.0, 1.25, 1.5, and 2.0), fibrin glue, and cyanoacrylate glue on porcine skin, measured 1 h post‐application. k) Bonding strengths of the A‐1.0 adhesive on various non‐biological substrates. l,m) Optical (l) and corresponding H&E‐stained histological images (m) of wounds treated with PBS, cyanoacrylate glue, and A‐1.25 adhesive over time. Reproduced with permission.^[^ ^51^ ^]^ Copyright 2021, American Chemical Society.

Emerging covalent bonding mechanisms based on diazirine‐mediated carbene insertion and isocyanate‐driven urea linkage formation are drawing attention as powerful alternatives to traditional strategies such as hydrogen bonding and catechol chemistry. A representative example of a diazirine‐based system is CaproGlu, a light‐curable tissue adhesive synthesized by grafting trifluoromethylphenyl diazirine (TPD) moieties onto biodegradable polycaprolactone triol (PCLT) (**Figure** **6**a).^[^ ^29^ ^]^ Upon exposure to UVA light, the diazirine groups broke down into extremely reactive carbene and diazoalkane intermediates. These intermediates readily insert into C─H, N─H, and O─H bonds on amino acid side chains in biological tissues, enabling covalent and nonspecific crosslinking to a variety of tissue substrates without the use of photoinitiators or co‐reagents. As illustrated in Figure 6b, CaproGlu demonstrated effective adhesion on hydrated rabbit liver tissues. Application of the liquid adhesive, followed by UVA irradiation, rapidly formed a conformal, viscoelastic biorubber film tightly bonded to the wet surface. Figure 6c revealed the kinetics of such a rapid transition from liquid to solid states. UV exposure instantly induced diazirine consumption and simultaneously formed diazoalkane and ether linkages, indicating carbene insertion into hydroxyl groups of PCLT. Together with controlling UVA dose, the incorporation of additives such as citric acid (CA) or hydroxyapatite (HA) could tune the mechanical properties of CaproGlu (Figure 6d). Increasing the UVA dose progressively enhanced the storage modulus in all formulations except pristine PCLT. Notably, formulations with 10 wt.% CA or HA (10 or 50 wt.%) exhibited significantly higher moduli, attributed to hygroscopicity and filler‐enabled toughening effects. This tunability allowed CaproGlu to be optimized for specific tissue types, including soft muscles and rigid bones. Figure 6e highlights the strong interfacial adhesion on both mineralized substrates and collagen‐rich soft tissues, as assessed by lap shear tests. Adhesion strengths exceeded 100 kPa in most cases, with particularly high values of ≈800 and ≈300 kPa on the interior and posterior bone surfaces, respectively—levels rarely attained by conventional adhesive mechanisms. This combination of rapid sealing and high adhesion strength can enable effective sealing in complex biological environments. Figure 6f presents the in vivo application of CaproGlu as a bioresorbable sealant for vascular anastomosis in a rabbit iliac artery model. Both partial (“half‐moon”) and full (“full‐moon”) arterial splices were treated using either traditional suturing or CaproGlu‐assisted repair. In the latter case, a primer layer of CaproGlu was first UVA‐cured onto the vessel wall, followed by the application of a 3D‐printed PCL mesh that was photochemically bonded in place using additional CaproGlu. This method enabled effective vessel approximation and hemostasis with minimal blood leakage. As shown in Figure 6g, histological analysis at 7 days revealed tissue integration and mild‐to‐moderate inflammation, comparable to conventional sutures. By 21 days post‐surgery, the CaproGlu had been completely resorbed, demonstrating its potential as a minimally invasive, photo‐activated adhesive platform for vascular repair without long‐term foreign body response. Here, the polymer backbone regulated the biodegradation behavior rather than the diazirine itself. Enzymatic cleavage and ester hydrolysis typically occurred in carriers based on polycaprolactone or gelatin (t_1_/_2_ ≈ 2–4 weeks in PBS, 37 °C), which resulted in almost complete resorption in vivo in 2–3 months. As bulk erosion continued, adhesion strength usually deteriorated after two weeks. On the other hand, persistent aromatic fragments should be avoided; the degradation products, which include aliphatic esters and amino acid derivatives, were biocompatible and metabolizable. Such degradation products were well tolerated, as demonstrated by rodent wound‐healing models, which show no signs of systemic toxicity or chronic inflammation.^[^ ^69^ ^]^

In addition to diazirine‐mediated systems, isocyanate‐based adhesives offer an efficient, bioorthogonal route to form robust covalent networks under physiological conditions. A compelling example is a solventless dual‐component bioadhesive system composed of two liquid precursors: hyperbranched polythioether (HBPTE) with terminal thiol (─SH) groups and polyethylene glycol functionalized with isocyanate groups (PEGNCO) (Figure 6h).^[^ ^51^ ^]^ The two‐step adhesive mechanism of this system in Figure 6i involves: First, strong interfacial adhesion was achieved via chemical bonding of isocyanate (─NCO) groups with nucleophilic residues (e.g., ─NH_2_ and ─OH) on tissue surfaces; Second, the cohesive strength of the adhesive bulk was driven by rapid and dense network formation via thiol‐isocyanate crosslinking. This synergistic combination enabled both immediate tissue anchoring and durable mechanical integrity. Figure 6j explored bonding performance depending on various [─NCO]/[─SH] molar ratios. The A‐1.25 formulation (1.25:1 ratio of isocyanate to thiol) demonstrated the highest lap shear adhesion strength on porcine skin, reaching ≈170 kPa after 1 h, which was over 13‐fold stronger than fibrin glue, and approaching the performance of commercial cyanoacrylate adhesives. This bonding performance resulted from an optimal balance between sufficient crosslinking density (for bulk cohesion) and adequate surface reactivity (for interfacial adhesion). Importantly, the HBPTE–PEGNCO system exhibited universal substrate compatibility, achieving strong adhesion not only on wet biological tissues but also on various engineering materials (Figure 6k). The A‐1.0 formulation (1:1 molar ratio) provided high bonding strengths across stainless steel (≈338 kPa), iron (≈420 kPa), glass (≈330 kPa), and common polymers such as polypropylene (PP), polyethylene terephthalate (PET), and polyphthalamide (PPA), indicating additional contributions from mechanical interlocking and intermolecular interactions (e.g., van der Waals forces) in the absence of covalent bonding.^[^ ^70^ ^]^ Figure 6i validated in vivo applicability and biocompatibility of the optimized A‐1.25 formulation using a full‐thickness skin incision model in Sprague Dawley rats. Compared to both saline‐treated controls and commercial cyanoacrylate glue (denoted as CA), the A‐1.25‐treated wounds exhibited faster closure rates and enhanced collagen deposition. Histological analysis in Figure 6m showed minimal inflammatory cell infiltration in A‐1.25‐treated tissues 14 days after surgery, while the cyanoacrylate group showed elevated immune cell presence (indicated as red arrows). Generally, urethane (carbamate) and urea linkages are hydrolytically labile and gradually degrade under physiological conditions. Poly(ester‐urethane) adhesives typically lose ≈30–50% of their mass within 6–8 weeks, accompanied by a proportional decrease in adhesion strength (e.g., from ≈60 to ≈25 kPa over 6 weeks).^[^ ^71^ ^]^ Polyurethane–urea adhesives degrade into aliphatic diols and amines, which are readily metabolized and exhibit low toxicity, in contrast to cyanoacrylates that release cytotoxic formaldehyde upon degradation. In vivo studies further indicate favorable biocompatibility with vital organs such as the kidneys, liver, and pancreas.^[^ ^51^, ^71^ ^]^

Beyond degradability and covalent bonding strength, the biomechanical safety limits of target tissues must also guide the design of diazirine‐ and urea‐linkage‐based adhesives. In shear or lap tests, these adhesives frequently show very high adhesion values (>50–100 kPa), which may be higher than the acceptable limits of fragile tissues like the pericardium, dura mater, or intestine, where failure stresses can be as low as 5–15 kPa. Vascular and muscular tissues, on the other hand, can tolerate ≈20–40 kPa, and mineralized or load‐bearing sites can safely support even higher adhesion (>100 kPa). This comparison highlights a key design challenge: balancing fluid‐tight sealing and mechanical stability with biomechanical safety. The tunability of diazirine and isocyanate chemistries, through curing dose, crosslinking density, or polymer backbone, provides opportunities to calibrate adhesion strength according to tissue type. Therefore, careful engineering of initial adhesion is necessary to meet tissue‐specific safety margins, even though degradability ensures safe long‐term resorption.

## 4 Conclusion and Perspectives

In this review, we introduced interfacial adhesion mechanisms, material systems, and biomedical applications of biodegradable bioadhesives, categorizing recent advances into five strategies: physical interlocking, hydrogen bonding, catechol chemistry, amine–carboxyl coupling, and diazirine or isocyanate‐based covalent linkages. Mechanically engineered structures, such as microtopographies and mesoporous particles, enhanced adhesion through topographic conformity and capillary effects. Hydrogen bonding‐based hydrogels offered reversible and dynamic adhesion with tunable mechanical and electrical properties. Catechol‐functionalized materials achieved robust bonding, particularly for underwater applications and sensor integration. Amine–carboxyl coupling provided rapid covalent sealing without external stimuli, while emerging chemistries, such as photoactivatable diazirine and urea‐based linkages, enabled spatiotemporal control and expanded substrate versatility.

Despite substantial progress, several challenges must be addressed to enable the clinical and commercial translation of biodegradable bioadhesives. First, underexplored adhesion mechanisms, including diazirine‐based carbene insertion and urea linkages, can offer distinctive advantages beyond traditional strategies. For example, silane‐based coupling, widely used in surface modification, holds potential for durable adhesion at bio‐inorganic and bio‐organic interfaces. The ability to generate siloxane bonds with hydroxylated surfaces enables integration with both soft tissues and rigid substrates such as bone or medical devices. Embedding silane functionalities within bioresorbable polymers could expand their utility in multifunctional adhesives tailored for hybrid tissue‐device applications. Additionally, metal–ligand coordination, inspired by biological systems such as mussels and marine worms, offers reversible and wet‐resistant adhesion with strong wet adhesion. Incorporation of biocompatible ions, e.g., Ca^2^⁺, Fe^3^⁺, into adhesive systems could facilitate tissue‐responsive interactions, especially in mineralized or vascular environments. Second, effective bioadhesive design must account for the diverse mechanical and physiological environments of tissues. Cardiac and intestinal tissues undergo continuous dynamic motions, while neural tissues are extremely soft and fragile. Skin represents an intermediate case with moderate stiffness and moisture, whereas bone is rigid and relatively dry. These variations in elasticity, moisture, and motion indicate that a single universal adhesive is unlikely to meet all biomedical needs, and significant mechanical mismatch between tissues and adhesives may lead to interfacial delamination, tearing, or even adverse biological responses. To tackle this challenge, compliant interface designs, such as modulus gradient tuning and double‐network structures (consisting of a rigid primary network and a soft, adhesive, energy‐dissipating secondary network), offer promising approaches to alleviate mismatch. In parallel, tissue‐specific customization, including adjustments in hydrophilic/hydrophobic balance, modulus, elasticity, and dynamic adhesion mechanisms, will be essential for creating stable and biocompatible interfaces tailored to each environment. Third, long‐term biocompatibility and degradation behavior remain insufficiently explored. While many studies confirmed acute cytocompatibility and short‐term in vivo performance, comprehensive evaluations of chronic inflammation, immunogenicity, and the metabolic fate of degradation byproducts are rare. Therefore, studies using large‐animal models and higher adhesive dosages are necessary to establish reliable biosafety profiles. Fourth, integrating “smart” functionalities can expand the utility of biodegradable bioadhesives. For example, stimuli‐responsive interactions, triggered by light, temperature, ultrasound, or magnetic fields, can enhance the precision of adhesive activation and disassembly. Such features are particularly relevant for minimally invasive procedures, where localization and timing of adhesive performance are critical, e.g., as endoscopic surgeries. Material design must be case‐specific, carefully balancing mechanical robustness/stretchability, degradation kinetics, and other application requirements. For implantable systems, prolonged adhesion under dynamic movements and seamless integration with surrounding biology are critical. In contrast, applications including wound dressings or degradable electronics may primarily consider rapid curing, on‐demand disintegration, and minimal foreign body response. Furthermore, stimuli‐responsive chemical bonds, such as redox‐, pH‐, or enzyme‐cleavable bonds, may allow adhesives to degrade in response to local tissue environments like inflammation, ischemia, or infection, and self‐reporting systems based on electrochemical, mechanochromic, or fluorescent signals might give physicians immediate feedback on adhesion integrity or failure indicators. Lastly, the successful clinical translation of biodegradable adhesives will require resolving key issues such as sterilization, long‐term storage stability, and rigorous regulatory approval. Conventional methods like autoclaving, ethylene oxide gas, and gamma irradiation can disrupt network architectures or degrade sensitive functional groups (e.g., NHS esters, diazirines), while hydrogel‐based formulations often face limited storage stability. In addition, extensive testing is essential for regulatory clearance, including ISO 10993 biocompatibility assessments, reproducible manufacturing, and large‐animal validation. In parallel, it is important to benchmark biodegradable adhesives against well‐established non‐biodegradable systems. Cyanoacrylates, epoxies, and PEG‐based sealants are already clinically approved for their rapid and durable bonding, yet their permanence may cause fibrosis, scarring, or foreign body reactions in delicate tissues. By contrast, biodegradable adhesives provide sufficient but transient adhesion during healing, followed by safe resorption without surgical removal. They are best suited for regenerative, pediatric, or resorbable device applications, whereas non‐degradables remain preferable for permanent fixation. It might be easier to comprehend their unique clinical role when we consider them as complementary tools rather than competitors.

Moving forward, interdisciplinary strategies that combine polymer chemistry, mechanobiology, tissue engineering, and electronic device fabrication will be essential. By aligning adhesive properties with the complexity of physiological environments and clinical needs, next‐generation biodegradable bioadhesives can evolve from static sealing materials into dynamic, intelligent platforms for surgical, therapeutic, and diagnostic innovation.

## Conflict of Interest

The authors declare no conflict of interest.
